# High-glucose induced toxicity in HK-2 cells can be alleviated by inhibition of miRNA-320c

**DOI:** 10.1080/0886022X.2022.2106874

**Published:** 2022-08-15

**Authors:** Yan Sun, Hai Qu, Qi Song, Yifan Shen, Lijuan Wang, Xiaohong Niu

**Affiliations:** a Heji Hospital Affiliated to Changzhi Medical College, Changzhi, China; b Department of General Surgery, Heji Hospital Affiliated to Changzhi Medical College, Changzhi, China

**Keywords:** miR-320c, PTEN, PI3K/AKT, HK-2, diabetic nephropathy

## Abstract

Diabetic nephropathy (DN) is a major healthcare challenge worldwide. MiRNAs exert a regulatory effect on the progress of DN. Our study proposed to investigate the miR-320c expression and its function on the pathogenesis of DN *in vitro*. The level of miR-320c in HK-2 cells was quantified by RT-qPCR. Cell morphology, invasion, and migration were observed by optical microscope, Transwell invasion assay, and scratch wound assay. Then, the levels of PTEN, α-SMA, vimentin, E-cadherin, p-PI3K, PI3K, AKT, and p-AKT were analyzed through western blotting. A Dual-luciferase reporter assay was conducted to explore the target relationship between miR-320c and PTEN. It was discovered that miR-320c was over-expressed in high glucose (HG)-treated HK-2 cells. Furthermore, inhibition of miR-320c could alleviate the epithelial-mesenchymal transition (EMT) of HG-induced HK-2 cells and retain the normal morphology of HK-2 cells. Additionally, the miR-320c inhibitor decreased the invasiveness and migration of HG-treated HK-2 cells. Next, the target gene of miR-320c, PTEN, was identified, and the function of miR-320c was reversed by down-regulation of PTEN. Finally, we found inhibition of miR-320c restrained the PI3K/AKT pathway. Therefore, inhibition of miR-320c could alleviate toxicity of HK-2 cells induced by HG *via* targeting PTEN and restraining the PI3K/AKT pathway, illustrating that miR-320c may act as a new biomarker in the diagnosis of DN.

## 1. Introduction

Diabetic nephropathy (DN) is lethal and a primary cause of renal failure in diabetic complications [1]. DN affected approximately one-third of diabetes patients, and it has become a notable global health problem [2]. The major causes of DN included renal hemodynamic changes, oxidative stress, and glucose metabolism disorder [3,4]. Nevertheless, its explicit molecular mechanism for developing effective therapies remained ambiguous.

MicroRNAs (miRNAs, miR) are a group of small RNA molecules that is non-coding and single-stranded and owned 18–22 nucleotides approximately [5]. Increasingly, recent evidence suggests miRNAs involvement in the occurrence and progression of DN [6–8]. For example, overexpression of miR-141 aggravated inflammation and facilitated apoptosis in mesangial cells treated with HG [9]. MiR-93 overexpression could restrain TGF-β1-induced epithelial-mesenchymal transition (EMT) and renal fibrosis by inhibiting Orai1 [10]. Based on these findings, miRNAs can potentially serve as therapeutic targets or diagnostic biomarkers in DN.

It has been reported that miR-320c is capable of inhibiting migratory potential in cervical cancer by targeting GABRP [11]. Besides, miR-320c, targeting CDK6, suppresses the proliferation, migration, and invasion of bladder cancer cells [12]. Moreover, researchers have identified miR-320c as a potential tumor suppressor miRNA as it is predicted to target multiple myeloma-associated oncogenes [13]. However, recent research suggests that the expression of miR-320c is elevated in DN patients compared with type 2 diabetes patients and healthy controls, suggesting that miR-320c overexpression is harmful to DN, but the underlying mechanism is unclear [14]. In the present study, the expression characteristic of miR-320c in HG-induced HK-2 cells and its possible molecular mechanism in DN were explored.

## 2. Materials and methods

### 2.1. Hk-2 cells authenticated

The human proximal tubule cell line (HK-2) was obtained from Procell (Wuhan, China). The HK-2 cells were tested and authenticated according to our previous work. DNA was extracted from HK-2 cells (1 × 10^6^ cells) by Chelex100, 20 STR loci and sex identification loci were amplified using the 21 CELLID system, PCR products were detected by ABI3130 × 1 genetic analyzer, and results were analyzed by GeneMapper IDX system, and compared with the database of ATCC, DSMZ, JCRB, and Cellosaurus. The STR results showed that the cells obtained from the cell bank are 100% matched to HK-2 cells. All experiments were performed with mycoplasma-free cells.

### 2.2. Cell culture and transfection

HK-2 cells were supplied with Dulbecco's modified Eagle medium (DMEM, Invitrogen, USA), supplemented with 10% fetal bovine serum (FBS, Sigma-Aldrich, USA). Cells were cultured at 37 °C in a CO_2_ incubator. First, to determine the optimal time for HG treatment, HK-2 cells were cultured in a serum-free cell culture medium (Invitrogen, USA) containing 45 mM glucose (HG, Sigma-Aldrich, USA) or 5.5 mM glucose (normal glucose, control), which had a purity ≥ 99.5% and was suitable for the culturing of mammalian cells for 6 h, 12 h, 24 h, and 48 h. Then, for the study, HK-2 cells were treated with HG for 24 h and transfected with small interfering RNA targeting PTEN (si-PTEN), miR-320c inhibitor and its negative control, inhibitor NC (Ribobio, China) at a final concentration of 50-100 nM. All transfections were conducted by Lipofectamine™ 2000 reagent (Invitrogen, USA) based on the manufacturer’s instructions.

### 2.3. Cell morphology observation and CCK-8 assay

After 48 h of HG induction, HK-2 cells were collected for cell smear preparation, and the morphology of HK-2 cells was observed under an ordinary optical microscope (Olympus CX41, Tokyo, Japan). The assay cell counting kit-8 (CCK-8) was used to detect the effect of HG or miR-320c inhibitor on HK-2 cells based on the manufacturer's instruction and measured the absorbance of each well at 450 nm.

### 2.4. Transwell invasion assay

Invasion assay was conducted by Transwell 24-well plates using filters of 8-μm diameter (Corning, NY, USA). Matrigel (BD, USA) and 4 °C pre-cooled serum-free medium were diluted at a ratio of 1:8 and 80 μl of the mixture were added to the upper chamber and incubated for 5 h at 37 °C. Then the lower chamber was supplied with a complete medium containing miR-320c inhibitor, inhibitor NC, or si-PTEN. After 48 h, the cells attached to the upper surface of the filter membranes were scrubbed with a cotton swab, and migrated cells at the lower surface were stained with 0.1% crystal violet at room temperature for 30 min. Then, 500 μl, 33% acetic acid was added, the membrane was immersed, and the solution was fully dissolved after 10 min of oscillation. The liquid from the 24-well plate was transferred to the 96-well plate and the number of invaded cells was observed using ImageJ software (National Institutes of Health, Bethesda, MD).

### 2.5. Scratch wound assay

For detection of the migration ability of miR-320c or PTEN on HK-2 cells, 2 × 10^5^ cells/well were cultured in the 6-well plate and incubated at 37 °C overnight. The monolayer was scratched by a tip and detached cells were removed by washing with a serum-free medium. Next, the cells were cultured in a complete medium supplemented with miR-320c inhibitor, inhibitor NC, or si-PTEN. HK-2 cells were photographed through the inverted microscope (TE2000, Nikon) at 0 h and 24 h post-wounding. The ImageJ analysis software (National Institutes of Health, Bethesda, MD, USA) was used to measure the migration distances.

### 2.6. Dual-luciferase assay

The HEK-293T cells which were seeded into the 48-well plates were treated with 5 pmol miR-320c inhibitor, inhibitor NC and 160 ng pSI-Check2-PTEN-3’UTR. After transfection for 48 h, the cells were collected and lysed. TheDual-Glo^®^ Luciferase Assay System (Promega, USA) was utilized to determine the luciferase reporter activities. The experiment was repeated at least three times. The values of each well were determined as the ratio of Renilla luciferase to Firefly luciferase.

### 2.7. Rt‑qPCR assay

Total RNA from HK-2 was extracted by TRIzol reagent based on the manufacturer's instructions (Invitrogen, USA). To quantify the expression of miR-320c. Primers are as follows. U6 forward primer: CTCGCTTCGGCAGCACA; U6 reverse primer: AACGCTTCACGAATTT -GCGT; miR-320c forward primer: AAAAGCAGGGAAGAGAGGGA. Cells were conducted by SYBR Green assay (Vazyme, China) based on the manufacturer’s protocol. U6 acted as a control and the 2^‑ΔΔCt^ method was used to quantify the relative expression level of miR-320c.

### 2.8. Western blot assay

To confirm the expression levels of E-cadherin, vimentin, α-SMA, PTEN, Akt, p-Akt, and PI3K, HK-2 cells were lysed with RIPA buffer (Beyotime, China). Lysates were separated on SDS-polyacrylamide gel and proteins were transferred to polyvinylidene difluoride (PVDF) membranes. Then, the membranes were blocked with 5% nonfat milk at room temperature for 1 h, and membranes were incubated with corresponding primary antibodies at 4 °C overnight following incubation with horseradish peroxidase-conjugated secondary antibody at room temperature for 1 h. Protein expressions were detected with high sensitivity ECL chemiluminescence detection kit (Vazyme, China). Using ImageJ software (National Institutes of Health, Bethesda, MD, USA), the band’s intensity was expressed as fold change by normalizing the data to the values of β-actin.

### 2.9. Statistical analysis

Statistical analysis was performed by SPSS22.0 and GraphPad Prism 9 software. Data were shown as means ± standard deviation (SD). One-way analysis of variance (ANOVA) or two-way ANOVA was used to compare the difference among multiple groups, however, the difference between the two groups was analyzed by the student t-test method. *p* < 0.05 was thought to be statistically significant.

## 3. Results

### 3.1. Mir-320c was up-regulated in the HG-induced HK-2 cells

**Figure 1.**
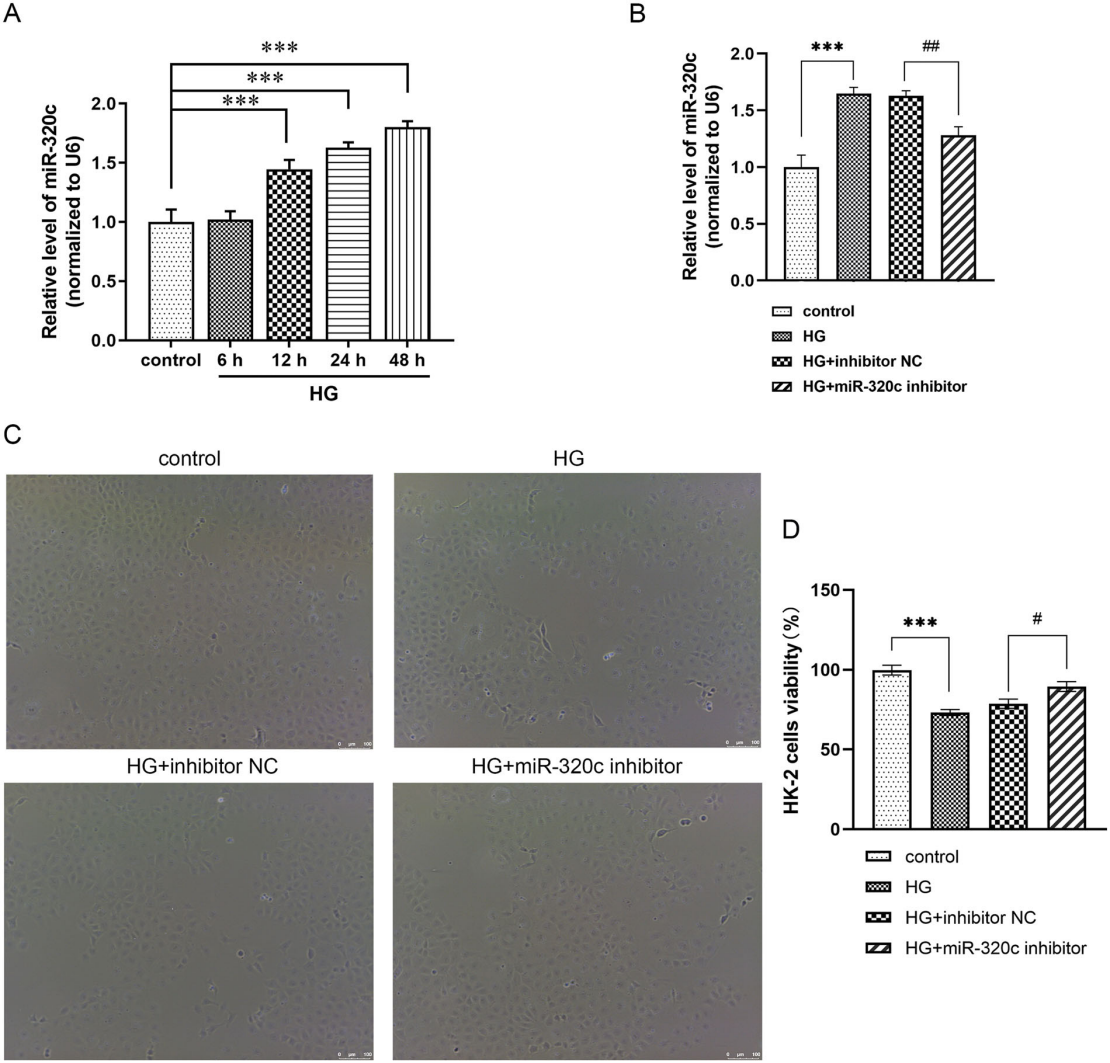
miR-320c was up-regulated in the HG-induced HK-2 cells and inhibition of it changed cell morphology of these cells. (A) The HK-2 cells were split into control group or HG group. The level of miR-320c was determined by RT-qPCR at 6 h, 12 h, 24 h, and 48 h. (B) HK-2 cells were transfected with miR-320c inhibitor or inhibitor NC and treated with HG for 24 h, and the level of miR-320c was quantified by RT-qPCR, U6 served as control. (C) The morphology of HK-2 cells in each group was observed by an ordinary optical microscope at a magnification of 100×. Scale bar = 100 μm. (D) CCK-8 was used to quantify the HK-2 cells viability. Bars represent the mean ± S.D from three independent experiments. Compared with the control group, ***p < 0.001; compared with the HG + inhibitor NC group, ^#^*p* < 0.05, ^##^*p* < 0.01.

For determining the expression feature of miR-320c in HK-2 cells treated with HG at different time points (6, 12, 24, and 48 h), we performed the following experiments. Results revealed that compared with the control group, the level of miR-320c was prominently enhanced in HK-2 cells by HG supplement at 12, 24, and 48 h in a time-dependent way (Figure 1(A)). In addition, miR-320c expression in cells transfected with miR-320c inhibitor and treated with HG for 24 h was decreased compared with the HG + inhibitor NC group, while it was increased by HG (Figure 1(B)). These data demonstrated that miR-320c was up-regulated in HG-induced HK-2 cells and miR-320c inhibitor could down-regulated the level of miR-320c. HK-2 cells growth as shown in (Figure 1(C)), and HK-2 cells viability as shown in (Figure 1(D)), indicated that HG inhibits the growth of HK-2 cells and miR-320c inhibitor relive the suppression that HG-induced.

### 3.2. Inhibition of miR-320c alleviated EMT of HG-induced HK-2 cells

**Figure 2.**
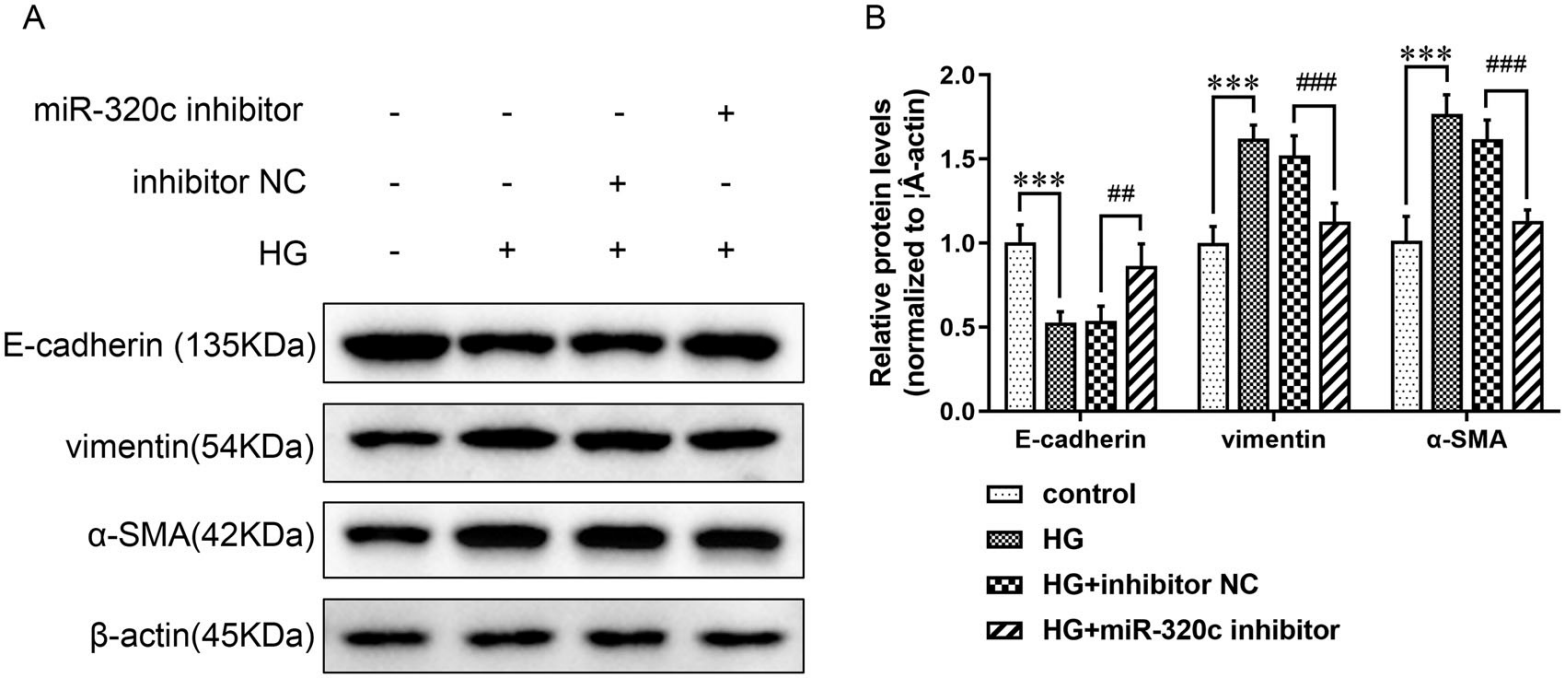
Inhibition of miR-320c alleviated EMT of HG-induced HK-2 cells. (A) HK-2 cells were transfected with miR-320c inhibitor or inhibitor NC and treated with HG, and the expressions of E-cadherin, vimentin, and α-SMA were analyzed by WB in each group. Β-actin served as control. (B) The relative protein levels of E-cadherin, vimentin, and α-SMA were quantified by ImageJ software. Bars represent the mean ± SD from three independent experiments. Compared with the control group, ***p < 0.001; compared with the HG + inhibitor NC group, ^##^*p* < 0.01, ^###^*p* < 0.001.

According to reports, EMT in renal tubular epithelial cells was critical to DN pathogenesis ^15^. Therefore, we explored whether miR-320c was related to EMT in HG-induced HK-2 cells. The data suggested that the levels of vimentin and α-SMA were obviously increased in the HG group compared with the control group, but cells treated with miR-320c inhibitor could partially down-regulated expressions of the two proteins compared with inhibitor NC. On the contrary, the level of E-cadherin presented an opposite trend (Figure 2(A,B)). These data suggested that down-regulating miR-320c could mitigate EMT in HK-2 cells treated with HG.

### 3.3. Inhibition of miR-320c reduced migration and invasion of HG-induced HK-2 cells

**Figure 3.**
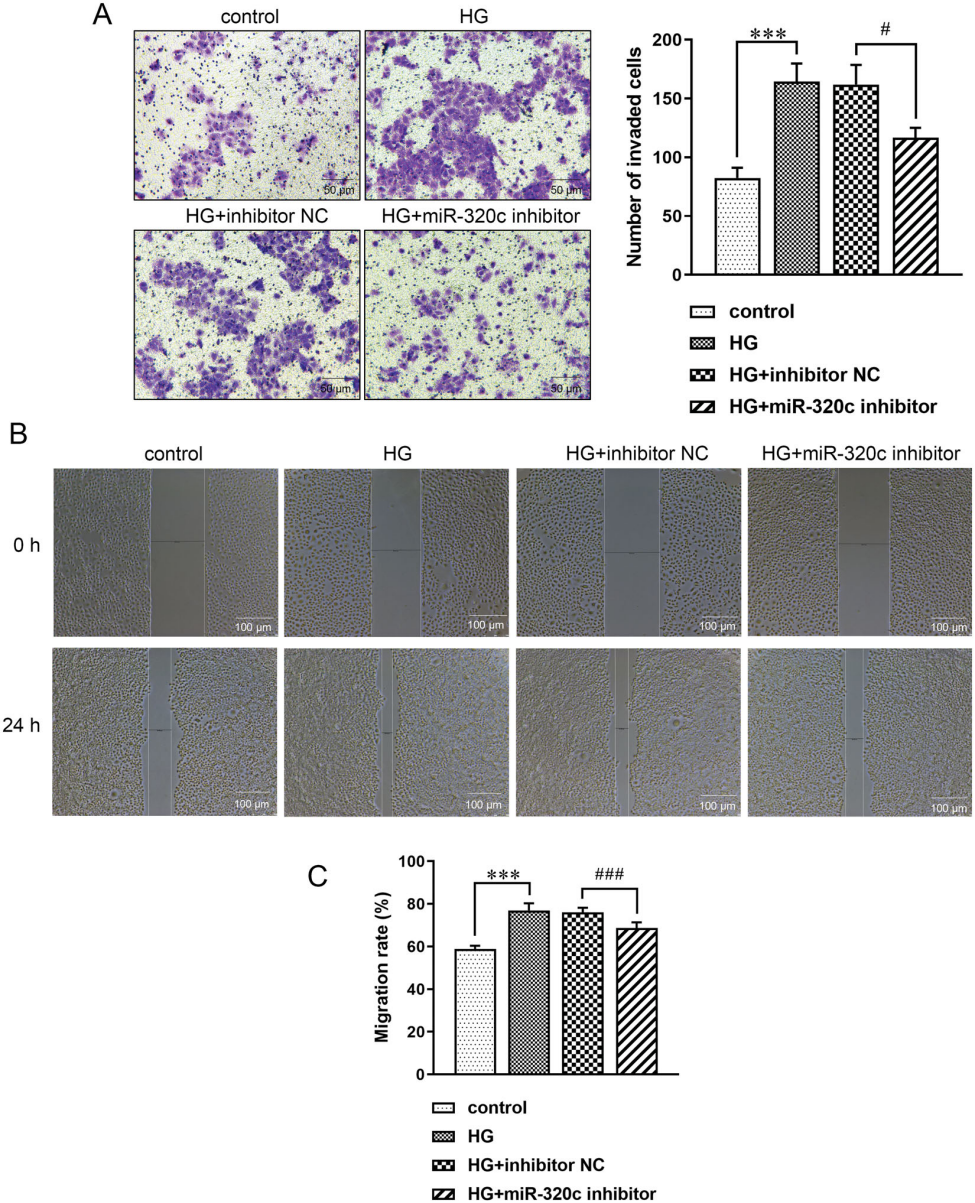
Inhibition of miR-320c reduced migration and invasion of HG-induced HK-2 cells. (A) Invasiveness of HK-2 cells treated with HG, miR-320c inhibitor, and inhibitor NC was demonstrated *via* Transwell invasion assay and quantified by the number of invaded cells. Under a microscope at a magnification of 200×, Scale bar = 50 μm (B) HK-2 cells were treated with HG, miR-320c inhibitor, and inhibitor NC. Scratch wound assay was utilized to measure the migration of HK-2 cells. Under a microscope at a magnification of 100×, Scale bar = 100 μm. (C) The migration of HK-2 cells was quantified by the migration rate in each group. Bars represent the mean ± SD from three independent experiments. Compared with the control group, ***p < 0.001; compared with the HG + inhibitor NC group, ^#^*p* < 0.05, ^###^*p* < 0.001.

A Transwell invasion assay was conducted for assessing the role of miR-320c on the invasiveness of HK-2 cells. Results exhibited that the number of invaded cells was markedly enhanced in the HG group, but the miR-320c inhibitor could significantly decrease the invasiveness of these cells (Figure 3(A)). Next, the role of miR-320c on HK-2 cell migration was detected *via* scratch wound assay, and the results suggested that the migration of HK-2 cells has no apparent difference among groups at the beginning. After 24 h, the migration of HK-2 cells treated with HG was prominently higher than the control group while miR-320c inhibitor treatment reversed this (Figure 3(B)), and this was illustrated by the significantly elevated or decreased migration rate in HG or HG + miR-320c group, respectively (Figure 3(C)). These data suggested that miR-320c reduced migration and invasion of HG-induced HK-2 cells.

### 3.4. Inhibition of miR-320c restrained the PI3K/AKT pathway in HG-induced HK-2 cells

**Figure 4.**
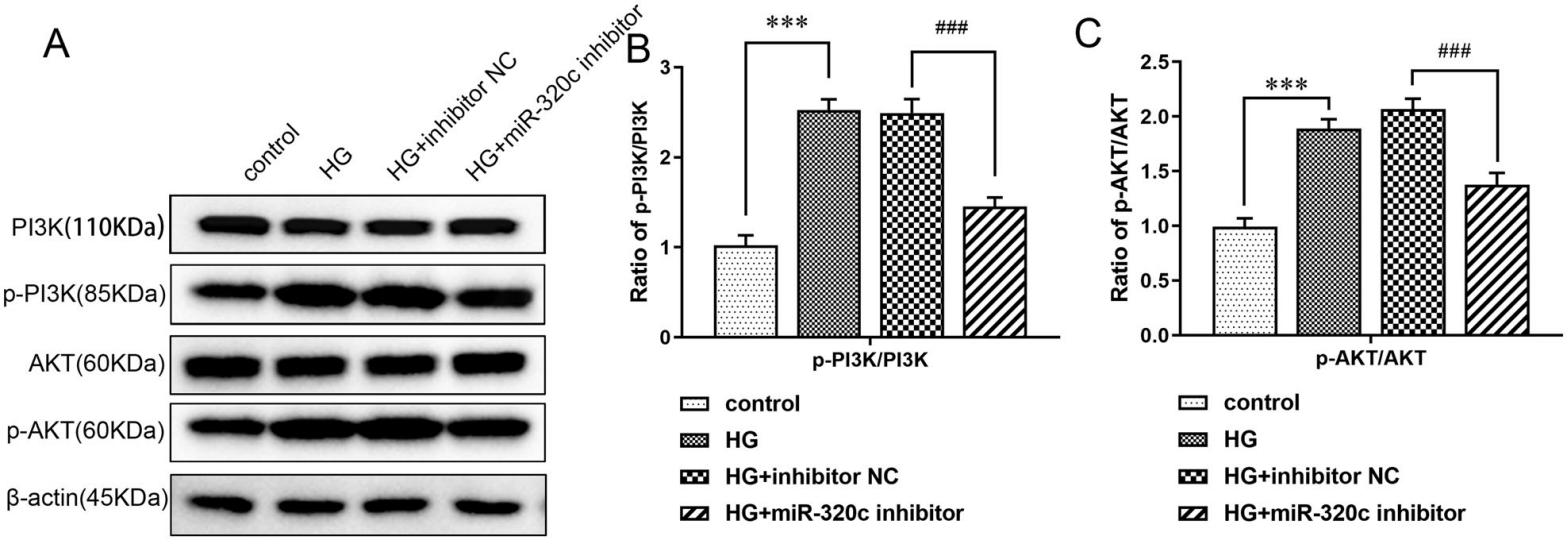
**Inhibition of miR-320c restrained the PI3K/AKT pathway in HG-induced HK-2 cells** (A) HK-2 cells were transfected with miR-320c inhibitor or inhibitor NC and treated with HG. The expressions of p-PI3K, PI3K, AKT, and p-AKT were analyzed by WB. (B) The ratio of p-PI3K/PI3K and (C)the ratio of p-AKT/AKT were quantified using ImageJ software. Bars represent the mean ± S.D from three independent experiments. Compared with the control group, ***p < 0.001; compared with the HG + inhibitor NC group, ^###^*p* < 0.001.

In HK-2 cells, we found that HG treatment could markedly enhance the ratio of p-PI3K/PI3K and p-AKT/AKT, suggesting that the PI3K/AKT pathway may be involved in the process of DN. However, compared with inhibitor NC, the expressions of these proteins were accordingly reduced in the miR-320c inhibitor group (Figure 4(A,B,C)). These observations implied the function of miR-320c may be connected with the PI3K/AKT signaling pathway.

### 3.5. PTEN was the direct target gene of miR-320c

**Figure 5.**
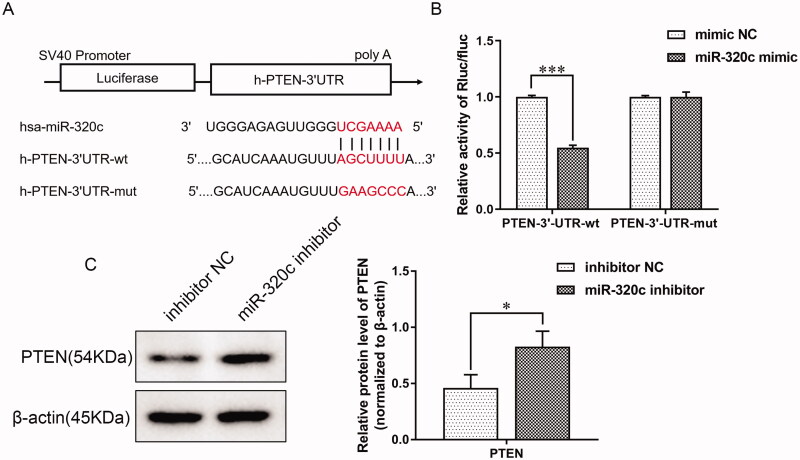
PTEN was the direct target gene of miR-320c. (A) The putative miR-320c binding sites in the PTEN sequence. (B) MiR-320c mimic or mimic NC and pSI-Check2-ATG7-3’UTR plasmid was co-transfected into HEK-293T cells. The relative firefly luciferase activity was determined after transfection for 48 h. (C) miR-320c inhibitor or inhibitor NC were transfected into HK-2 cells and the protein expression of PTEN was conducted by WB and quantified by ImageJ software, with β-actin as a loading control. Bars represent the mean ± SD from three independent experiments. *p < 0.05 and ***p < 0.0001.

To figure out the underlying mechanism of miR-320c, we predicted that PTEN was its possible target gene by the online tool Targetscan (Figure 5(A)). For verifying the relationship between miR-320c and the 3ˊUTR of the PTEN gene, we constructed the wide type (wt) or mutation (mut) firefly luciferase reporter containing the 3ˊUTR of PTEN and conducted the luciferase assay. Results suggested that miR‑320c mimic could prominently reduce the luciferase activity of cells transfected with PTEN 3ˊUTR‑wt compared with the mimic NC group, but no significant difference was observed in PTEN 3'UTR‑mut groups, suggesting that miR‑320c may directly target PTEN (Figure 5(B)). Furthermore, PTEN expression was revealed to be up-regulated at the protein level (Figure 5(C)) after miR‑320c inhibitor treatment. These results suggested a direct interaction between miR-320c and the 3ˊUTR of the PTEN gene.

### 3.6. Down-regulation of PTEN reversed the function of miR-320c on HG-induced HK-2 cells

**Figure 6.**
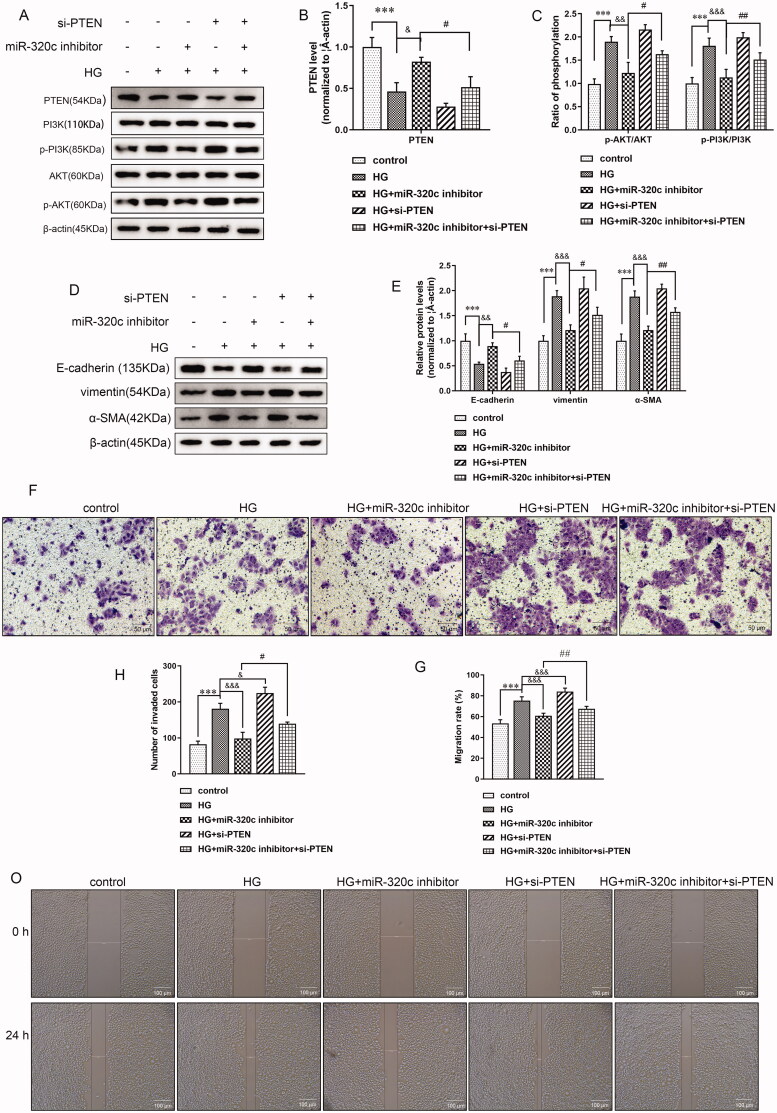
Down-regulation of PTEN reversed the function of miR-320c on HG-induced HK-2 cells. HK-2 cells were transfected with miR-320c inhibitor, si-PTEN, or both and treated with HG, and the expressions of (A) PTEN, p-PI3K, PI3K, AKT, p-AKT (D) E-cadherin, vimentin, and α-SMA were analyzed by WB. (B) PTEN level, (C)p-AKT/AKT and p-PI3K/PI3K ratio, and € E-cadherin, vimentin, and α-SMA were quantified by ImageJ software. Β-actin served as control. (F) Transwell invasion assay was performed, scale bar = 50 μm and (H) numbers of invaded cells were quantified. (O) The migration ability of HK-2 cells was measured by scratch wound assay, and (G) migration ratio was quantified by the migration rate in each group. Scale bar = 100 μm. Bars represent the mean ± SD from three independent experiments. Compared with the control group, ***p < 0.001; compared with the HG group, ^&^*p* < 0.05, ^&&^*p* < 0.01, ^&&&^*p* < 0.001, compared with the HG + miR-320c inhibitor, ^#^*p* < 0.05, ^##^*p* < 0.01.

For confirmation of whether miR-320c regulated DN *via* PTEN, we treated HG-induced HK-2 cells with si-PTEN, miR-320c inhibitor, or both. Firstly, the ratio of p-PI3K/PI3K and the ratio of p-AKT/AKT were significantly decreased compared with the HG group (Figure 6(A,C)), but si-PTEN treatment could markedly up-r the expression of PI3egulateK/AKT pathway (Figure 6(A,B)). Next, the results of WB indicated that the expression levels of vimentin and α-SMA were obviously decreased after miR-320c inhibitor treatment compared with the HG group, but si-PTEN could partially up-regulate expressions of the two proteins compared with miR-320c inhibitor alone. On the contrary, the level of E-cadherin presented an opposite trend (Figure 6(D,E)). Besides, the transwell invasion assay exhibited that the number of invaded cells was markedly down-regulated in HG-treated HK-2 cells and miR-320c inhibitor compared with HG alone, but si-PTEN addition could significantly promote the invasiveness of these cells (Figure 6(F,G)). Lastly, scratch wound assay revealed that the migration of HK-2 cells has no apparent difference among groups at the beginning. After 24 h, the migration of HK-2 cells treated with HG and miR-320c inhibitor was lower than the HG group while si-PTEN treatment prominently reversed this, and these were illustrated by the significantly decreased or elevated migration rate in HG + miR-320c inhibitor or HG + miR-320c inhibitor + si-PTEN group, respectively (Figure 6(H,O)). The trend was following the former results. The result of the above demonstrated that inhibition of miR-320c and PTEN simultaneously had the reverse effect on DN compared with down-regulation of miR-320c alone, demonstrating that the effect of miR-320c was related to regulating PTEN.

## 4. Discussion

In this study, we demonstrated that miR-320c was up-regulated in HK-2 cells treated with HG compared with normal HK-2 cells. Additionally, inhibition of miR-320c could restrain EMT, invasion, and migration of HK-2 cells induced by HG. Next, we revealed that PTEN was a regulatory target gene of miR-320c, and the function of miR-320c could be reversed by PTEN. Finally, the miR-320c inhibitor had a negative effect on the activation of the PI3K/AKT signaling pathway. Our study may provide a new therapeutic target, miR-320c, for the diagnosis and treatment of DN.

Nowadays, the function of miRNAs had become a promising topic in the development and progression of various diseases [16]. It has been reported that the abnormal expression of miRNAs was related to autoimmune and cardiovascular diseases, even a variety of cancers, and DN [17–19]. For instance, miR-21 inhibitors could restrain the development of renal fibrosis and EMT, while enhancing the structure and function of kidneys in DN [20,21]. And miR-34a-5p was up-regulated in HG-induced HK-2 cells and overexpression of it could aggravate tubulointerstitial fibrosis by targeting SIRT1 [22]. In diabetes, overexpression of miR-25 could reduce hypertension and revert kidney changes of DN [23]. Importantly, researchers have demonstrated that miR-320c revealed the highest expression level in DN patients, suggesting that miR-320c may serve as a novel target marker for diagnosis of type II DN [24]. Therefore, we aimed to illustrate the regulative role and molecular mechanism of miR-320c in DN. In this study, we found that the level of miR-320c in HG-induced HK-2 cells was markedly higher than in normal HK-2 cells. Then, we synthesized the miR-320c inhibitor to explore its function *in vitro*. The data showed that miR-320c inhibition could retain the cobblestone-like cube shape of HK-2 cells, suggesting that miR-320c may be harmful to DN.

EMT was identified as one of the pathogenesis in the development and progression of renal tubulointerstitial fibrosis [25]. Renal biopsies from diseased kidneys laid the foundation for understanding the pathology of EMT, which suggested the ratio of cells experiencing transition correlated to the degree of interstitial fibrosis and the level of serum creatinine both [26]. The evidence that EMT occurred *in vivo* had been demonstrated in some chronic kidney diseases, including DN [27,28]. Although tubulointerstitial fibrosis may also appear in these early stages, the accumulation of tubulointerstitial fibrosis was often accompanied by disease progression and was related to the gradual decline of renal function [29]. Epithelial cells transferred from the epithelial compartment to the mesenchyme through EMT obtained a complete mesenchymal phenotype and were characterized by decreased expressions of E-cadherin or increased expressions of α-smooth muscle actin (α-SMA) and vimentin among epithelial cells [30,31]. Up-regulation of vimentin caused a reorganization of the cortical actin cytoskeleton into a cytoplasmic and basal network of intermediate filaments [32]. Then, the cell motility and the formation of new membrane protrusions were promoted by this transition to a mesenchymal state. Finally, expression of metalloproteinase and enhanced cell protrusions resulted in cell migration, invasive behavior, and extra cellular matrix degradation [33,34]. In other words, the development of EMT was accompanied by enhanced migrated and invasive abilities [35]. In the present study, we found that miR-320c inhibitor treatment prominently down-regulated the expression of mesenchymal cell markers α-SMA and vimentin, and increased E-cadherin expression in HG-induced HK-2 cells, suggesting the suppression of EMT. In addition, transwell invasion assay and scratch wound assay exhibited that the number of invaded cells and migration rate were markedly increased in HK-2 cells treated with HG, but miR-320c inhibitor could significantly decrease the invasiveness and migration of these cells. These data further illustrated the inhibiting EMT effect of miR-320c in HG-induced HK-2 cells.

Phosphatase and tensin homolog (PTEN), identified as a dual-function lipid and protein phosphatase, could regulate a variety of cellular processes, including metabolism, cell growth, and migration [36,37]. Previous studies have shown that PTEN was connected with renal fibrosis in DN [38,39]. Therefore, PTEN was not only referred to as a tumor suppressor, but it also played an anti-fibrotic role. Researchers have demonstrated that HG treatment could inhibit the expression of PTEN, which induced podocyte EMT through restraining the PI3K/Akt pathway [40]. So far, only a few miRNAs performed their biological effects by targeting PTEN in DN. For instance, miR-21 accelerated renal fibrosis in DN by targeting PTEN and SMAD7 [38]. In this study, we demonstrated that miR-320c could directly target PTEN, and inhibition of PTEN could revert the function of miR-320c inhibitor on EMT, invasion, and migration of HK-2 cells treated by HG. Therefore, we concluded that inhibition of miR-320c could alleviate EMT of HG-treated HK-2 cells by enhancing PTEN expression.

PI3K/Akt signaling pathway was involved in many biological processes, including regulating metabolism, cell proliferation and differentiation, and other physiological processes [41]. At present, researchers have revealed that the imbalance of the PI3K/Akt pathway in DN was compactly associated with fibrosis. Overexpression of TGF-β1 and activation of Akt were involved in the formation of renal tubular EMT induced by HG [42]. Researchers found that an inhibitor of Akt, Ginkgo biloba extract, might alleviate renal interstitial fibrosis in DN [43]. Besides, in diabetic mice, PTEN alleviated the ECM deposition to alleviate the renal damage by restraining the Akt and CTGF expressions [39]. Based on these, we showed that HG treatment could increase the expressions of p-PI3K/PI3K and p-AKT/AKT, suggesting that PI3K/AKT was activated in the process of DN. However, the expressions of these proteins were accordingly decreased after miR-320c inhibitor treatment. These observations suggested that the mechanism of the miR-320c inhibitor was restraining PI3K/AKT signaling pathway.

## 5. Conclusion

In short, this study demonstrated that miR-320c was up-regulated in HG-induced HK-2 cells, and the miR-320c inhibitor could suppress EMT, invasion, and migration of HG-induced HK-2 cells *via* regulating PTEN. Besides, the miR-320c inhibitor could restrain the expressions of p-PI3K/PI3K and p-AKT/AKT. Therefore, the miR-320c inhibitor may prevent the occurrence of DN by inhibiting PTEN and PI3K/AKT signaling pathways. These findings further demonstrated that miR-320c might serve as a novel target for DN treatment.

## Data Availability

The datasets used and/or analyzed during the present study are available from the corresponding author on reasonable request.
